# Thirdhand Tobacco Smoke: Emerging Evidence and Arguments for a Multidisciplinary Research Agenda

**DOI:** 10.1289/ehp.1103500

**Published:** 2011-05-31

**Authors:** Georg E. Matt, Penelope J. E. Quintana, Hugo Destaillats, Lara A. Gundel, Mohamad Sleiman, Brett C. Singer, Peyton Jacob, Neal Benowitz, Jonathan P. Winickoff, Virender Rehan, Prue Talbot, Suzaynn Schick, Jonathan Samet, Yinsheng Wang, Bo Hang, Manuela Martins-Green, James F. Pankow, Melbourne F. Hovell

**Affiliations:** 1 Department of Psychology, and; 2 Graduate School of Public Health, San Diego State University, San Diego, California, USA; 3 Indoor Environment Department, Lawrence Berkeley National Laboratory, Berkeley, California USA; 4 San Francisco General Hospital Medical Center, University of California San Francisco, San Francisco, California, USA; 5 Center for Child and Adolescent Health Policy, Massachusetts General Hospital, Boston, Massachusetts, USA; 6 David Geffen School of Medicine, University of California Los Angeles, Los Angeles, California, USA; 7 Stem Cell Center, University of California Riverside, Riverside, California, USA; 8 Keck School of Medicine, University of Southern California, Los Angeles, California, USA; 9 Environmental Toxicology, University of California Riverside, Riverside, California, USA; 10 Department of Cancer and DNA Damage Responses, Lawrence Berkeley National Laboratory, Berkeley, California, USA; 11 University of California Riverside, Department of Cell Biology and Neuroscience, Riverside, California, USA; 12 Portland State University, Civil and Environmental Engineering and Chemistry, Portland, Oregon, USA

**Keywords:** aggregate exposures, biomarkers, cumulative exposure, exposure, housing, nicotine, policy, secondhand smoke, tobacco smoke

## Abstract

Background: There is broad consensus regarding the health impact of tobacco use and secondhand smoke exposure, yet considerable ambiguity exists about the nature and consequences of thirdhand smoke (THS).

Objectives: We introduce definitions of THS and THS exposure and review recent findings about constituents, indoor sorption–desorption dynamics, and transformations of THS; distribution and persistence of THS in residential settings; implications for pathways of exposure; potential clinical significance and health effects; and behavioral and policy issues that affect and are affected by THS.

Discussion: Physical and chemical transformations of tobacco smoke pollutants take place over time scales ranging from seconds to months and include the creation of secondary pollutants that in some cases are more toxic (e.g., tobacco-specific nitrosamines). THS persists in real-world residential settings in the air, dust, and surfaces and is associated with elevated levels of nicotine on hands and cotinine in urine of nonsmokers residing in homes previously occupied by smokers. Much still needs to be learned about the chemistry, exposure, toxicology, health risks, and policy implications of THS.

Conclusion: The existing evidence on THS provides strong support for pursuing a programmatic research agenda to close gaps in our current understanding of the chemistry, exposure, toxicology, and health effects of THS, as well as its behavioral, economic, and sociocultural considerations and consequences. Such a research agenda is necessary to illuminate the role of THS in existing and future tobacco control efforts to decrease smoking initiation and smoking levels, to increase cessation attempts and sustained cessation, and to reduce the cumulative effects of tobacco use on morbidity and mortality.

In this article, we aim to clarify ambiguities and misunderstandings in the scientific community regarding thirdhand smoke (THS), also known as residual or aged tobacco smoke. The significance of THS in the broader context of tobacco control efforts and its specific role in causing, contributing, moderating, or mediating tobacco-related illnesses have been questioned. To paraphrase an anonymous reviewer of an earlier manuscript on THS: THS is probably no more than a trivial nuisance, no worse than spilled coffee. This skepticism is in contrast to the positions taken by the public health community on issues of tobacco control in general and involuntary exposure to tobacco smoke in particular. The 2006 U.S. Surgeon General’s Report on the health consequences of involuntary exposure to tobacco smoke concluded: “The scientific evidence indicates that there is no risk-free level of exposure to secondhand smoke” (U.S. Department of Health and Human Services 2006). If decades of scientific research support the conclusion that there is no risk-free level of exposure to the potent mixture of carcinogens, irritants, and other toxicants in secondhand smoke (SHS), the composition, prevalence, and distribution of THS and the acute and cumulative exposure to those compounds among nonsmokers should be examined before we declare THS pollution and exposure a mere nuisance. We must better understand the role of THS exposure of people, particularly children, to the components of tobacco smoke. The implications of this exposure for disease mechanisms and their moderators and the THS-related acute and long-term risks of disease and premature mortality must be examined. Finally, we should consider the degree to which such understanding can inform and perhaps transform tobacco control policies, which would allow nonsmokers, their families, and the public to make more informed decisions about living in THS-polluted environments and to help smokers to better understand the risks their smoking brings to others. Perhaps a better understanding of THS and the associated risks to nonsmokers, stricter norms and attitudes, and economic and social contingencies will motivate nonsmokers not to start and prompt addicted smokers to quit. We propose that the “bench to bedside to population” approach of translational research will be useful in guiding research on THS and in fostering translation of findings to protect public health (National Institutes of Health 2009).

We review the emerging evidence on THS and outline the case for an interdisciplinary research effort. The existing evidence provides strong support for pursuing a programmatic research agenda on THS to fill important gaps in our current understanding of the short- and long-term effects of involuntary exposure to tobacco smoke. We begin with brief definitions of THS and THS exposure. We then present a review of recent findings on the chemistry of THS, its persistence in indoor environments, implications for pathways of exposure and health effects, and behavioral and policy issues that affect and are affected by THS. We conclude with recommendations for interdisciplinary research efforts to address the gap in knowledge of the biological mechanisms of toxicity on cellular and molecular levels, as well as relevant behavioral, economic, and sociocultural considerations and consequences.

## What Is Thirdhand Smoke? How Is it Different from Secondhand Smoke?

SHS is a mixture of the sidestream smoke (i.e., smoke emitted from the burning cigarette, pipe, or cigar) and the mainstream smoke exhaled from the lungs of smokers. SHS contains more than 4,000 chemicals, many of which are known or suspected contributors to adverse health effects. These chemicals include ammonia, acrolein, carbon monoxide, formaldehyde, hydrogen cyanide, nicotine, nitrogen oxides, polycyclic aromatic hydrocarbons (PAHs), and sulfur dioxide, as well as other chemicals that are eye and respiratory irritants, mutagens, carcinogens, and cardiovascular and reproductive toxicants (U.S. Department of Health and Human Services 2006).

THS consists of residual tobacco smoke pollutants that remain on surfaces and in dust after tobacco has been smoked, are re-emitted into the gas phase, or react with oxidants and other compounds in the environment to yield secondary pollutants. The constituents of THS identified to date include nicotine, 3-ethenylpyridine (3-EP), phenol, cresols, naphthalene, formaldehyde, and tobacco- specific nitrosamines (including some not found in freshly emitted tobacco smoke) (Destaillats et al. 2006; Singer et al. 2002, 2003, 2004; Sleiman et al. 2010b).

SHS exposure results from the involuntary inhalation of sidestream and exhaled mainstream smoke. In contrast, THS exposure results from the involuntary inhalation, ingestion, or dermal uptake of THS pollutants in the air, in dust, and on surfaces. Such exposure includes inhalation exposure to compounds re-emitted into the air from indoor surfaces and particles resuspended from deposits, and dermal and ingestion exposure to compounds partially derived from cigarette smoke and resulting particles that have settled, deposited, and accumulated on surfaces.

Although the term THS is relatively new (New York Times 2009; Szabo 2006), the chemical aging of tobacco smoke, the evidence THS leaves behind in indoor environments (e.g., cigarette butts, unpleasant odor, smelly clothes), and its aversive impact on nonsmokers have long been recognized. We favor the term “thirdhand smoke,” rather than alternative terms such as aged tobacco smoke or residual SHS, to stress that THS is the legacy of tobacco smoke, it evolves from SHS and, similar to SHS, it leads to involuntary exposure to tobacco smoke pollutants. Although it is important to distinguish SHS from THS because of significant chemical, toxicological, and behavioral differences, SHS and THS are closely related and coexist during the early period of THS formation and in contaminated environments in which smoking takes place episodically.

Based on our definitions of SHS and THS, total tobacco smoke exposure is the cumulative involuntary exposure to tobacco smoke pollutants during and after the time in which cigarettes are smoked. The exposure risk does not end when a cigarette has been extinguished and may persist in the absence of further smoking, because THS pollutants, trapped and deposited on surfaces and in dust, persist in environments in which smoking took place at some earlier points in time.

## Constituents, Sorption–Desorption Dynamics, and Transformations of THS

Some of the pollutants present in SHS remain principally in the gas phase and can be removed by ventilation, but a significant fraction adheres to indoor surfaces and persists for a longer time. Complex physicochemical transformations of those compounds take place after smoking (i.e., aging) that affect both short- and long-term exposure patterns of nonsmokers. Aging processes include chemical reactions of residual components of tobacco smoke deposited on indoor surfaces and may be influenced by pollutant transport between different indoor media (e.g., the deposition into deep reservoirs such as the gypsum core of wallboard panels). Physical and chemical transformations of tobacco smoke pollutants take place simultaneously over time scales that range from a few seconds to several weeks or months after their initial release during smoking. During an initial period of up to a few hours immediately after smoking, SHS and THS exposure coexist, with the latter becoming predominant once SHS is removed by ventilation.

*Indoor sorption–desorption dynamics.* Indoor surface:volume ratios are often in the range of 1–10 m^2^/m^3^, which are much larger than typical outdoor ratios (Knutson et al. 1992). Partitioning of volatile organic compounds (VOCs) and semivolatile organic compounds (SVOCs) to surfaces is a key mediator of human exposure to indoor pollutants. Building materials and furnishings often operate as sinks, reservoirs, or sources for these chemicals. The affinity of a VOC to building products (such as carpet, gypsum board, upholstery, flooring material, and acoustic tiles) is inversely proportional to the vapor pressure of the compound and is affected by specific molecular interactions and competition with water vapor (Won et al. 2001). Tobacco smoke contains both VOCs and SVOCs; the latter partition between aerosol particles and the gas phase according to Junge-Pankow model predictions (Liang and Pankow 1996; Pankow et al. 1994). Partitioning must include the indoor materials, as described by Weschler and Nazaroff (2008, 2010). Nicotine is one of the major SVOCs released in large amounts during smoking (1–3 mg/cigarette) (Singer et al. 2003). Other authors have reported higher amounts; the 1999 Massachusetts Benchmark Study reported nicotine levels in sidestream smoke ranging from 2.2 to 5.3 mg/cigarette, depending on cigarette brand (Borgerding et al. 2000). Nicotine room-temperature vapor pressure (0.04 mmHg) is between three and four orders of magnitude lower than that of indoor VOCs such as toluene (22 mmHg) or benzene (100 mmHg).

Sorption and desorption have been monitored in realistic settings by carrying out experiments in real indoor environments or in room-sized environmental chambers. For example, Singer et al. (2002, 2003) studied nicotine absorption dynamics in a room-sized 50-m^3^ chamber furnished with typical materials (wallboard, carpet, draperies, and furniture) that they exposed to tobacco smoke generated by machine-smoking. Exposure-relevant emission factors that account for sorptive uptake and re-emission have been determined for short-term (1 day) and long-term (1 month) periods for 26 gas-phase organic compounds present in tobacco smoke. Analytes included volatile aldehydes (formaldehyde, acrolein), aromatic hydrocarbons (benzene, toluene, naphthalene), nicotine, and tobacco-related amines (pyridine, 3-EP). The emission factor of each individual compound was influenced by sorption and re-emission from indoor surfaces and materials. For each analyte, sorptive losses (i.e., transfer from the gas phase to material surfaces) were found to be highest at the highest level of furnishing (i.e., when more effective surface area was available) and for lower room ventilation rates (i.e., higher pollutant residence times). Losses were more marked for the less-volatile chemicals, and they were particularly remarkable for nicotine. In a month-long cyclic smoking study, after an initial accumulation period of ~10 days, re-emission of accumulated nicotine from indoor surfaces became a source of gas-phase nicotine equal in strength to smoking (Singer et al. 2003). In subsequent experiments using the same chamber (Singer et al. 2004), pure chemicals were released by flash evaporation and allowed to partition between gas phase and indoor surfaces. Several tobacco smoke constituents (nicotine, ethenylpyridine, methyl naphthalenes, ortho-cresol) readily sorbed to chamber surfaces, with nicotine having the highest affinity for surfaces. Nicotine was almost completely removed from the gas phase and deposited on indoor surfaces, whereas most other chemicals showed more moderate partitioning behavior. The strong sorptive tendency of nicotine implies that indoor surfaces in environments where smoking is habitual can be loaded with large amounts of this alkaloid and other related THS components, creating a hidden reservoir of THS constituents that could be re-emitted long after the cessation of active smoking.

Spectroscopic evidence suggests that amines adsorb predominantly in a protonated state in the presence of moisture (Destaillats et al. 2007; Ongwandee et al. 2007). Sorptive interactions of nicotine and other tobacco alkaloids are strongly influenced by the presence of other common airborne acids and bases, such as carbon dioxide (CO_2_) and ammonia (NH_3_), respectively, that are often present at high concentrations indoors. In bench-scale studies, the sorptive capacity of common materials such as carpet and wallboard toward trimethylamine, a model amine, increased in the presence of CO_2_ and decreased in the presence of NH_3_ as a consequence of the enhancing protonation capacity of CO_2_ (acid) and the competition with NH_3_ (base) (Ongwandee et al. 2005; Ongwandee and Morrison 2008).

**Figure 1:**
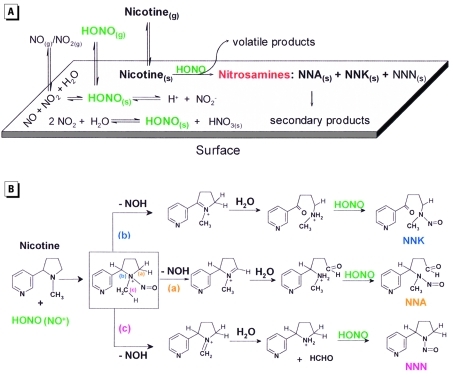
Physical-chemical processes of nicotine reactions with nitrous acid on indoor surfaces. (*A*) Illustration of surface-mediated nitrosation of nicotine. (*B*) Proposed mechanism for the formation of TSNAs. Adapted from Sleiman et al. (2010b). Abbreviations: (a), proposed mechanism for formation of NNA; (b), proposed mechanism for formation of NNK; (c), proposed mechanism for the formation of NNN; (g), gas phase; HCHO, formaldehyde; (s), on surface; secondary products are those created by indoor chemical reactions from primary tobacco smoke products (e.g., NNK from nicotine).

*Indoor chemical transformations.* Reactions driven by oxygenated and nitrogenated atmospheric species are the source of indoor secondary pollutants of potential toxicological relevance (Morrison 2008). A recent study identified the formation of carcinogenic tobacco-specific nitrosamines (TSNAs) from the reaction of adsorbed nicotine with nitrous acid (HONO) (Sleiman et al. 2010b). HONO is typically produced indoors by combustion sources and heterogeneous conversion of atmospheric nitrogen oxides. Nicotine adsorbed to a model surface showed high reactivity toward HONO, leading to the formation of three TSNAs: 1-(*N*-methyl-*N*-nitrosamino)-1-(3-pyridinyl)-4-butanal (NNA), 4-(methylnitrosamino)-1-(3-pyridinyl)-1-butanone (NNK), and *N*-nitroso nornicotine (NNN). The structures of these compounds as well as their mechanisms of formation are shown in Figure 1 (adapted from Sleiman et al. 2010b). NNA, which is not present in freshly emitted tobacco smoke, was the predominant TSNA. Because of their low vapor pressures, these TSNAs are likely associated with indoor surfaces and dust. In addition to TSNAs, nitrosation of nicotine generated low levels of *N*-nitrosopyrrolidine (a carcinogenic volatile nitrosamine) and various other multifunctional by-products.

Ozone and related atmospheric oxidants [hydroxyl radical and hydrogen peroxide (H_2_O_2_)] may generate oxidized products by reaction with some of the tobacco smoke components that remain sorbed to indoor surfaces. Thus, some of the respiratory symptoms associated with tobacco smoke may originate not from directly emitted air pollutants, but from volatile by-products that have low thresholds for eye, skin, and upper respiratory tract irritation (Destaillats et al. 2006; Singer et al. 2006).

The atmospheric lifetime of ozone is long enough to allow for its transport to the indoor environment, where it reacts at rates often higher than typical ventilation removal rates, leading to typical indoor/outdoor ratios between 0.2 and 0.7 (Weschler 2000). Typical indoor ozone levels in most settings are ≤ 20 ppb by volume (ppbV). However, much higher ozone levels may be generated using devices marketed as air purifiers and often used to remove tobacco odors (Boeniger 1995; Hubbard et al. 2005).

**Figure 2:**
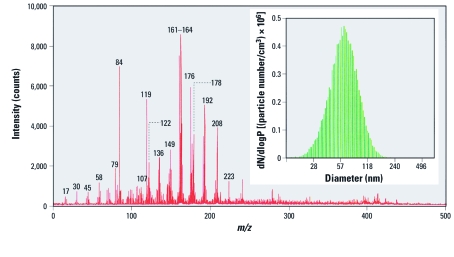
Mass spectrum and size distribution of secondary organic aerosol generated during nicotine reaction with ozone. Adapted from Sleiman et al. (2010a) with permission from Elsevier. dN/dlogP is the normalized particle number per size range.

**Figure 3:**
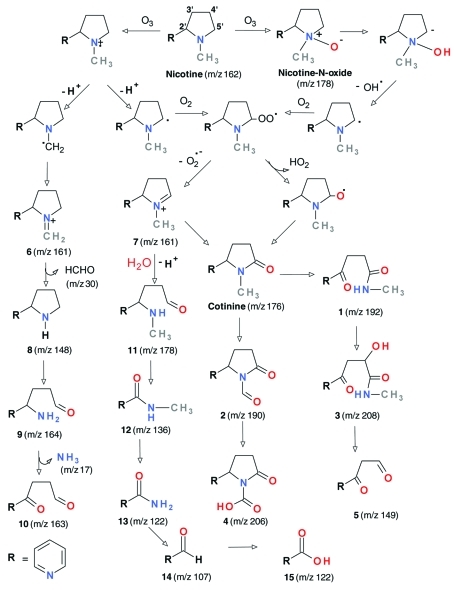
Reaction products and proposed pathways (shown by arrows) for nicotine reactions with ozone. Reprinted from Sleiman et al. (2010a) with permission from Elsevier. *m/z*****is the mass to charge ratio used to interpret mass spectral data.

The reaction of ozone with VOCs emitted during smoking was studied in a room-sized chamber (Shaughnesy et al. 2001). Ozone reacted rapidly with unsaturated VOCs such as isoprene, pyrrole, and styrene but was relatively inert toward aromatic hydrocarbons. The main by-products were volatile aldehydes, which included formaldehyde, acetaldehyde, and benzaldehyde. Although amine ozonation is typically slow in the gas phase, sorption of nicotine to indoor surfaces can extend its indoor residence time and make it more available for ozonation (Petrick et al. 2010). The reactivity of nicotine sorbed to model surfaces toward ozone was evaluated in laboratory experiments; formaldehyde, *N*-methyl formamide, myosmine, ethyl pyridyl ketone, nicotinaldehyde, and cotinine were formed and were re-emitted into the gas phase (Destaillats et al. 2006). Ozone reactions with nicotine or with SHS also formed ultrafine particles, as shown in Figure 2, in which several multifunctional oxidized species with high asthma hazard index (Jarvis et al. 2005) values were identified. Figure 3 illustrates the molecular structures of the identified nicotine oxidation by-products and their pathways of formation.

## Prevalence and Persistence of THS in Real-World Indoor Environments

The chemistry and physics of tobacco combustion in indoor environments suggest that some gas- and particle-phase THS compounds can remain for extended periods in all indoor environments in which tobacco smoke has been produced (Destaillats et al. 2006, 2007; Matt et al. 2004, 2008a, 2011; Singer et al. 2002, 2004). The persistence of THS in real-world residential settings has been demonstrated based on nicotine and 3-EP concentrations in air, dust, and surfaces in the days, weeks, and months after the last smoking has taken place. Further support comes from quantitative measurements of ultrafine tobacco smoke particles resuspended after their deposition on household surfaces (Becquemin et al. 2010).

Increased or persistent gas-phase air nicotine levels are indicative of a reservoir of sorbed nicotine in these environments. Nicotine levels in dust and on surfaces are proportional to THS matter that has deposited and accumulated on indoor surfaces, including coffee tables, bed frames, cabinets, doors, and walls. Nicotine in dust also represents THS trapped in carpets, upholstery, curtains, pillows, mattresses, and similar materials. In addition, PAHs, known human carcinogens associated with tobacco smoke and other combustion sources, have been demonstrated to accumulate in house dust from homes of smokers (Hoh E, personal communication). Hein et al. (1991) were the first to report elevated levels of nicotine in house dust that was collected in the homes of smokers and to point out the positive association of nicotine with smoking level. Nicotine also has been found to contaminate private homes of smokers in which smoking bans have been implemented (Matt et al. 2004) and the homes of nonsmokers that were formerly occupied by smokers (Matt et al. 2011).

The automobile cabin is another enclosed microenvironment with a high surface:volume ratio, where sorption of tobacco pollutants may lead to long-term contamination and substantial exposure of nonsmokers. In a recent study, cars of smokers who did not impose smoking bans exhibited high levels of nicotine on surfaces such as on dashboards, in dust, and in cabin air, with mean values of 8.6 μg/m^2^, 19.5 μg/g, and 740 ng/m^3^, respectively. These values were measured several hours or days after smoking took place and were significantly higher than were those from cars where smoking bans were implemented and from cars belonging to nonsmokers (Matt et al. 2008a). Nicotine also remained in used cars sold by smokers with and without car smoking bans and in rental cars (Matt et al. 2008a). Fortmann et al. (2010) found that smokers can lower THS contamination in their cars by reducing or by stopping smoking. They also reported that commonly used cleaning (e.g., vacuuming, wiping) and ventilation methods were unsuccessful in significantly lowering nicotine contamination.

*Implications for exposure.* The presence of THS compounds in the air, in dust, and on surfaces of indoor environments creates potential exposure routes through inhalation, ingestion, and dermal transfer. These pathways are likely to be relevant for children living in homes in which adults smoke, even if smoking occurs at times or in rooms when no children are present. Infants and young children are more likely to be at risk of THS exposure than are adults because they typically spend more time indoors, are in proximity to and engage in greater activity in areas where dust collects and may be resuspended (e.g., carpets on the floor), and they insert nonfood items in their mouths more frequently than do adults (U.S. Environmental Protection Agency 2008). It is estimated that infants and young children are 100 times more sensitive than adults to pollutants in house dust because of such factors as increased respiration relative to body size and immature metabolic capacity (Roberts et al. 2009). For other environmental toxicants such as lead, pesticides, allergens, endotoxins, and flame retardants, house dust has been reported to be the main route of exposure for infants and young children (U.S. Environmental Protection Agency 1997, 2008). Homes of smokers present an ongoing risk of THS exposure to children who live in the home in addition to SHS exposure they already receive when around a smoking adult. In addition, involuntary exposure of children of nonsmokers may occur when they unknowingly come in contact with THS in a polluted environment.

Indoor environments that frequently change ownership or occupancy present the highest risk of involuntary exposure to THS pollution for occupants. Such environments include rental apartments, condominiums and houses, hotel rooms, and rental and used cars. The increased risk of THS exposure in these environments is the result of two factors. First, these environments are often private spaces in which public smoking bans do not apply or private smoking bans are poorly implemented or monitored. Second, because smoking prevalence among adults is 10–25% in the United States (9.8% in Utah and 25.6% in Kentucky and West Virginia) (Centers for Disease Control and Prevention 2010), the probability that one or more smokers occupied and smoked in these environments is high. After only five occupancies, the probability that one or more smokers were among the occupants is 41% given a smoking prevalence of 0.10 and 76% given a prevalence of 0.25.

Although much THS appears to be stored in dust and on surfaces in a polluted environment, THS is not constrained to the physical space in which tobacco was smoked. Recognizable as stale tobacco smoke, THS is trapped on the clothes of smokers and nonsmokers who were exposed to SHS. Most important, THS is detectable on the hands of smokers beyond the environment in which they smoked (Matt et al. 2004, 2011), and smokers may spread THS pollutants to other persons (e.g., their infants) and other objects (e.g., toys, food).

When nonsmokers touch polluted surfaces in smoking environments, they sample pollutants via their hands. For example, Matt et al. (2011) recently demonstrated that THS is detectable in dust and on surfaces of apartments occupied by smokers and on the hands of nonsmokers who moved into these apartments more than 2 months after smokers moved out.

THS is a special concern in multiunit housing where smoking is permitted (Kraev et al. 2009). Tobacco smoke can move along air ducts, through wall and floor cracks, through elevator shafts, and along plumbing and electrical routes to contaminate units on other floors far removed from the smoking area (Spengler 1999). Tobacco smoke exposure in public housing is particularly troubling, because it disproportionately afflicts disadvantaged and vulnerable populations (Winickoff et al. 2009). In 2008–2009, 32% of households in public housing included elderly persons, 35% disabled persons, and 41% children (U.S. Department of Housing and Urban Development 2010). In a study of > 5,000 children in the United States, those who lived in multiunit housing apartments where their parents did not allow smoking had 140% higher serum cotinine levels than did children who lived in detached housing (Wilson et al. 2011).

In summary, the available evidence on THS pollution of indoor environments shows that THS is ubiquitous and pervasive wherever tobacco has been smoked. Its presence in air and dust and on surfaces allows for multiple exposure routes, and THS creates special risks for nonsmokers who spend time indoors in proximity to polluted surfaces. Infants and children are especially vulnerable, because of their increased exposure and increased sensitivity to pollutants, as are persons with limited mobility and populations that spend time in multiunit housing and spaces with frequent changes in occupancy.

## Potential Clinical Significance and Public Health Implications of THS

Currently, assessing the independent health risks of THS is premature because of the lack of evidence on clinical outcomes. The characterization of health risks attributable to THS will require applying new knowledge from cell and molecular biology, conducting clinical trials (e.g., randomized trials of health outcomes as a consequence of reduced exposure), and gathering objective evidence of THS contamination and biomarkers to confirm individual exposure and relating these data to outcomes (e.g., morbidity or premature mortality associated with THS). Clinical significance, however, must take into account the impact of THS in the broader context of tobacco control efforts to prevent and reduce smoking behavior.

*Potential health risks.* THS exposes people to mixtures of chemical compounds in gas phase and particulate phase similar to those contained in mainstream smoke and SHS, as well as to additional products of surface reactions involving tobacco smoke constituents. Because THS and SHS differ in the composition and distribution of pollutants and in exposure profiles, a simple quantitative comparison of pollutant concentrations is not possible. For instance, air nicotine levels of indoor environments in which active smoking takes place are excellent markers of SHS pollution and correlate well with SHS exposure as measured by urine cotinine levels (Jaakkola and Jaakkola 1997). Air nicotine levels, however, are not likely to be the best indicator of THS pollution, and pollutant levels of nicotine and other compounds on surfaces and in dust must also be considered. Urine cotinine levels are likely to poorly estimate exposure to THS constituents other than nicotine, including those that deposit on surfaces and in dust in proportions independent of nicotine.

Compared with SHS and active smoking, existing evidence suggests that exposure to THS involves very different time profiles of exposure (i.e., low-level cumulative exposure over long periods vs. repeated exposures to high levels over short intervals), different pollutant concentrations in different media (i.e., surfaces and dust vs. primarily air), novel pollutants not found in SHS, and different relative contributions of exposure routes (i.e., inhalation vs. dermal vs. ingestion) (Jaakkola and Jaakkola 1997). Consequently, health risks of THS may include some of those of SHS and active smoking as well as new ones not yet directly associated with tobacco smoke.

Human exposure to constituents of THS has not been well characterized, and it is therefore premature to assess the health risk of THS. Given this caveat, one can consider how some of the known THS components could affect human health. The chemicals that mediate adverse health consequences can be considered in categories such as irritants, carcinogens, and mutagens (e.g., TSNAs, PAHs, heavy metals, nicotine).

Nicotine plays multiple roles in carcinogenesis through inhibition of apoptosis and cell proliferation (Catassi et al. 2008; Wright et al. 1993; Zhou et al. 2010). It is known to affect oxidative stress and to have adverse effects on brain and lung development in children (Zhou et al. 2010). Nicotine may have adverse effects on vascular function and might promote inflammation (Wittebole et al. 2007). Because nicotine and other THS constituents may be transformed into new toxicants (Sleiman et al. 2010a, 2010b), concerns about potential health risks of THS must include compounds created through secondary reactions.

An important question is how many of the known carcinogens identified by the International Agency for Research on Cancer (IARC) that are found in mainstream and sidestream smoke are continuously or intermittently present in THS (IARC 2004). TSNAs, such as NNK, are potent lung carcinogens, and some TSNAs form from nicotine on indoor surfaces through chemical reactions with ambient nitrous acid (Hecht 2003). See Burton (2011) for an initial effort to quantify the potential exposure to NNA and NNK via dermal transfer. PAHs in tobacco smoke, particularly benzo[*a*]pyrene, are also carcinogenic (IARC 2004). Particles and oxidant gases produce free radical species and oxidant injury that can promote inflammation, affect immune function, and activate thrombotic mechanisms (Hamade et al. 2008; van Eeden et al. 2005). Oxidant and irritant gases can trigger allergic symptoms and asthma (Dworski 2000).

Comprehensive assessment of risks of THS will require characterization of levels of THS constituents in the environment, analysis of their cytotoxicity and genotoxicity *in vitro* and in animal models, measurement of human exposure based on validated biomarkers, and, eventually, epidemiologic studies of the association of THS exposure with morbidity and mortality.

Risk assessment will require the development of biomarkers of THS exposure. A logical initial focus for a selective biomarker might be metabolites of NNA, because NNA is the major TSNA formed from the reaction of nicotine and nitrous acid and has not been found in tobacco smoke. Likely metabolites are *iso*-NNAL [1-(*N*-methyl-*N*-nitrosamino)-1-(3-pyridinyl)-4-butanol) and iso-NNAC (4-(*N*-methyl-*N*-nitrosamino)-4-(3-pyridinyl)-butanoic acid], which might be measurable in urine. NNA or other substances derived from it might be suitable as markers of THS in dust or surfaces.

Risk assessments will benefit from careful consideration of sensitive populations (e.g., young children, medically compromised persons) and at-risk environments (e.g., low-income housing). Because of the immature stage of their biological and behavioral development, the level of exposure and health risks are likely to be greatest for young children who are in direct contact with polluted surfaces and house dust.

*Broader clinical and public health consequences of THS on tobacco control efforts.* Even though THS is a dynamic mixture of chemical compounds, it is important to remember that it is a consequence of smoking behavior, which is a modifiable human activity with well-understood harmful health outcomes. It is in this context that public awareness of THS, aversion to stale tobacco odor, and beliefs about THS take on clinical significance beyond any specific health effects of THS still to be demonstrated. For instance, knowledge about THS could be used clinically to encourage home and car smoking bans among individuals and to promote cessation. In one of the first studies on attitudes about THS, controlling for known confounders including SHS beliefs, respondents were asked whether they agreed that “smoking in a room today could cause harm to infants and children tomorrow” (Winickoff et al. 2009). Those who agreed were more than twice as likely to have had a strict home smoking ban than were those who disagreed with the statement. Those who were uncertain about the harmful effects were also more likely to have a strict home smoking ban (Winickoff et al. 2009).

## Policy Implications of THS for Overall Tobacco Control

Although it is premature to formulate public policies in response to potential THS health risks, it is important to note that numerous voluntary private policies have emerged over the past 10 years targeting THS. Major international, national, and local hotels (e.g., Marriot, Westin) and car rental companies (e.g., Avis, Enterprise, Hertz) have adopted complete or partial smoking bans to protect nonsmokers from the effects of lingering tobacco smoke. These policies grew out of complaints and concerns about unpleasant odor, respiratory symptoms, and eye irritation among hotel guests and customers of rental cars. Similar consumer preferences for smoke-free environments are also noticeable in the used car and real estate markets. Research conducted in southern California has shown that used cars of smokers were valued 8–9% lower than were the equivalent priced cars owned by nonsmokers (Matt et al. 2008b), and rental apartments remained vacant longer and required higher maintenance costs (Matt et al. 2011) when they were occupied by smokers rather than nonsmokers.

In the absence of definitive scientific evidence on health risks of THS, how did these policies and consumer preferences develop? We believe that the distinct unpleasant odor of stale tobacco smoke and acute respiratory and eye symptoms played a critical role, alerting consumers to a tobacco-polluted environment. This explanation is consistent with Junker et al. (2001) who demonstrated odor detection thresholds lower by three or more orders of magnitude than previously suggested for acceptable indoor conditions (> 19,000 m^3^/cigarette). Eye and nasal irritations were observed at levels one order of magnitude lower than previously thought, corresponding to a fresh air dilution volume of > 3,000 m^3^/cigarette. For comparison, a 1,000-square-foot apartment in the United States has a volume of < 300 m^3^. The practical significance of odor thresholds is captured by a popular saying in the real estate and used car markets: “If you can smell it, you can’t sell it.”

Odor thresholds and health symptoms by themselves, however, do not explain the recent emergence of THS policies and market place responses. We believe that consumer knowledge of the health effects of tobacco use, changing norms, expectations, and attitudes toward tobacco smoke exposure empowered consumers to express their dislike, request a nonsmoking hotel room, ask for repairs and cleaning, and negotiate a lower price. Such a marketplace response to consumer demands shows that when the norm and expectation are a smoke-free apartment, hotel room, or car, it is not only desirable but profitable for private businesses to establish smoke-free policies.

Although the emergence of smoke-free policies in the private sector appears to be a response to consumer demand, norms, and expectations, success of these policies in protecting nonsmokers is not at all certain. Whereas voluntary policies do not follow common standards for detecting THS pollutants, training employees, monitoring implementation, and enforcing compliance, public policies can introduce shared standards and direct attention to the neediest instead of the noisiest. Although consumer complaints about THS and demand for smoke-free environments provide an excellent starting point, we currently lack full understanding of how to promote or temper cultural demands for protection from THS exposure. We lack coordinated efforts to educate, reinforce, and strengthen norms toward establishing and maintaining 100% smoke-free environments should the toxicology and epidemiology justify such action. Public health policies regarding THS can emerge as an extension of current efforts to protect vulnerable nonsmokers from SHS as part of a coordinating tobacco control strategy toward completely smoke-free environments.

## Recommendations for an Interdisciplinary Research Agenda on THS

The following summarizes directions and recommendations for an interdisciplinary research agenda on THS. Consistent with the risk assessment framework introduced by the National Research Council (2009), the proposed agenda addresses issues surrounding hazard identification, dose–response assessment, exposure assessment, and risk characterization. We propose, however, to go beyond assessing the unique adverse health effects of THS components and to consider the role of THS as part of a broader tobacco control strategy. The goal of this programmatic agenda is to connect basic and applied research on risk assessment with research to prevent and reduce tobacco use, exposure to tobacco smoke pollutants, and tobacco-induced diseases.

*Chemistry of THS.* There is a considerable body of research on the chemistry of main and sidestream tobacco smoke and the agents that cause tobacco-induced diseases. However, much remains to be learned about the formation of new compounds by THS components through aging and interaction with environmental oxidants such as ozone, oxides of nitrogen and related compounds from both outdoor and indoor sources, and their relative proportion over the aging period. These processes need to be studied over days, weeks, and months in the presence and the absence of further smoking. The following research needs arise from the work to date and seem particularly relevant to us:

- Characterize as completely as possible the chemistry of THS; identify toxic and potentially toxic substances; examine how THS differs from SHS.
- Identify mechanisms of THS formation, reactive species, and reaction pathways.
- Examine how THS deposits and accumulates in dust, surfaces, and air, and how chemical mechanisms compare in these different media.
- Explore the interaction of THS with environmental oxidants such as air pollution and continued smoking through controlled lab studies and observational field studies of THS in actual indoor environments with typical smoking, cleaning, ventilation, and use patterns.
- Develop, test, and validate tracers of THS pollutants at different stages of aging (e.g., NNA) and for different media (dust, surface, air).

*Exposure assessment.* The assessment of exposure to THS has to consider smoking behavior that generates tobacco smoke; the environment that becomes contaminated with tobacco smoke pollutants; the behavior of smokers and nonsmokers in a polluted environment that brings them in contact with the pollutants; multiple pathways and time profiles over which exposure takes place; and efforts to protect an environment from pollutants and behaviors to prevent exposure from occurring. Because of the importance of dust and surfaces in the accumulation of THS, infants and children are most at risk of higher THS exposures as a result of their increased contact with dusts and surfaces and their close association with adults. Although there is a considerable body of literature on the exposure to tobacco smoke pollutants among active and passive smokers, little is currently known, for instance, about how different smoking patterns contribute to the accumulation of THS pollutants; how pollutants accumulate in different media; the relative effectiveness of different strategies to protect an environment from the accumulation of THS; and the relative importance of different pathways and profiles of exposure in different populations. We believe that the following topics require special attention in experimental and epidemiological research:

- Develop, test, validate biomarkers of exposure to different THS pollutants and different stages of THS aging, especially biomarkers suitable for use in children.
- Investigate association between smoking behavior, tracers for THS pollutants in air, dust, surface, and biomarkers of THS exposure.
- Compare indoor spaces with ongoing active smoking (SHS and THS) and indoor spaces that transitioned from smoker to smoke-free (aging THS).
- Examine occupational exposure risks (e.g., hospitality, delivery truck drivers).
- Conduct controlled human exposure experiments as a way of testing and validating biomarkers of exposure to THS.
- Evaluate the relative contribution of different exposure pathways in different settings and populations, such as in young children and in low-income households with other concomitant exposures such as traffic-related pollutants.
- Survey indoor environments (home, car, hotels, etc.) for THS tracers when exposed to environmental oxidants and investigate whether surrogate measure of oxidant levels (presence of gas stove in home, proximity to a busy street) can predict formation of more toxic THS components.
- Investigate THS pollution levels as a function of smoking behavior, cleaning, ventilation, activity patterns, household appliance, environments by season (outdoor temperature, weather, climate), income, socioeconomic status.
- Investigate effectiveness of different efforts to prevent THS pollution and exposure (e.g., smoking restrictions, cleanup and remediation).
- Investigate exposure disparities and environmental justice issues in THS exposures, especially in relation to low-income housing.

*Toxicology and health effects.* Although there is a growing body of research on the toxicology and health effects of tobacco smoke and SHS (i.e., dose–response assessment and risk characterization), currently very little is known about the potential and actual health effects attributable to THS. To achieve a better understanding of health effects attributable to THS, future experimental and epidemiological research should

- Develop biomarkers for disease or tissue damage caused by THS components.
- Study *in vitro* metabolism, toxicology, and genotoxic potential of THS components, especially compounds formed through aging and oxidant reactions.
- Carry out *in vivo* metabolism and toxicology studies of the most toxic compounds.
- Evaluate the toxicology of different exposure pathways (inhalation, dermal transfer, ingestion) and exposure profiles (acute and chronic, cumulative and single) and exposure during sensitive periods, such as infancy.
- Evaluate risk in medically compromised populations: respiratory and pulmonary; immune system; prenatal and neonates; at-risk groups, such as infants, children, and pregnant women, by environment (e.g., nonsmokers living with smokers, children cared for by smokers and in the homes of smokers); effects of exposure reduction in high-risk populations.
- Discriminate effects attributable to THS from those of SHS exposure and active smoking.
- Identify smoking behaviors and environments particularly hazardous to specific populations.

*Tobacco-related norms, preferences, and behaviors.* Our review suggests that concerns associated with THS shape behaviors and attitudes of individuals, local policies, and marketing strategies targeting consumers. We know little currently about how concerns related to THS emerge and evolve, how they shape behaviors of smokers and nonsmokers, and how they can be leveraged to reduce smoking and SHS and THS exposure. The following topics seem particularly worthwhile:

- Research on the nature, origins, and pervasiveness of THS awareness, concerns, attitudes, and norms.
- Quasi-experimental and observational research on how the evolution of tobacco-related norms and their gradual change affect smoking, SHS, and THS practices and policies.
- Research on the relationship between THS awareness, attitudes, norms, and their expression in consumer preferences and behavior.
- Intervention trials on how best to conduct health education and promotion campaigns to influence norms and expectations, adopt stricter bans, and reduce smoking behavior.
- Focused THS education campaigns and interventions to affect the valuation of smoke-free environments (real estate; cars; child home care).
- Development of counseling and coaching interventions for medically vulnerable populations to address disparities and to provide a more sensitive and immediate test of possible health benefits from intervention.

*Tobacco control policies: protecting nonsmokers from tobacco smoke pollutants.* Our review indicates that public awareness and consumer preference have given rise to a range of policies at local levels and in private enterprises. Although they are evidence of interest in and demand for further control of smoking behavior, the impact and limitations of these emerging policies are not well understood. The following research areas can help contribute important evidence for developing and implementing effective public and private policies to protect nonsmokers from THS and to reduce tobacco use overall.

- Studying the effectiveness of emerging local ordinances, corporate policies, and private bans and rules.
- Examining policy loopholes, vulnerable population, and critical environments informed by basic and clinical research.
- Investigating the need for better occupational exposure protection (e.g., hospitality industries, delivery truck drivers).
- Connecting policy efforts at the local, regional, and state levels and in personal, public, work, school, and business domains.
- Working with consumer organizations to incorporate preferences about smoke-free environments in informal and formal norms, property valuation, and standards for monitoring and compliance.

## Conclusion

The emerging evidence on THS suggests important new directions for understanding the long-term consequences of tobacco use and for preventing and reducing tobacco use. Although it is premature to trivialize or dramatize the significance of THS, the existing evidence provides strong support for pursuing a programmatic research agenda to fill important gaps in our current understanding of the chemistry, toxicology, pollution, exposure, clinical significance, and policy implications of THS. Such a research program is necessary to illuminate the role of THS in existing and future tobacco control efforts to decrease smoking initiation and smoking levels, to increase cessation attempts and sustained cessation, and to reduce the cumulative effects of tobacco use on morbidity and mortality.
